# Network pharmacology‒based analysis of marine cyanobacteria derived bioactive compounds for application to Alzheimer’s disease

**DOI:** 10.3389/fphar.2023.1249632

**Published:** 2023-10-19

**Authors:** Rui Xie, Feng Chen, Yixuan Ma, Wen Hu, Qiang Zheng, Jinguo Cao, Yi Wu

**Affiliations:** ^1^ Key Laboratory of Prevention and Treatment of Cardiovascular and Cerebrovascular Diseases of Ministry of Education, Gannan Medical University, Ganzhou, China; ^2^ Department of Pediatric Surgery, First Affiliated Hospital of Gannan Medical University, Ganzhou, China; ^3^ State Key Laboratory of Marine Environmental Science, Institute of Marine Microbes and Ecospheres, Xiamen University, Xiamen, China; ^4^ College of Ocean and Earth Sciences, Xiamen University, Xiamen, China; ^5^ School of Basic Medical Sciences, Gannan Medical University, Ganzhou, China

**Keywords:** cyanobacteria, marine Synechococcus sp., gas chromatography-mass spectrometry, volatile organic compounds, Alzheimer’s disease, network pharmacology

## Abstract

In recent years, the Alzheimer’s disease (AD) epidemic has become one of the largest global healthcare crises. Besides, the available systemic therapies for AD are still inadequate. Due to the insufficient therapeutic options, new treatment strategies are urgently needed to achieve a satisfactory therapeutic effect. Marine bio-resources have been accepted as one of the most economically viable and sustainable sources with potential applications for drug discovery and development. In this study, a marine cyanobacteria–*Synechococcus* sp. XM-24 was selected as the object of research, to systematically investigate its therapeutic potential mechanisms for AD. The major active compounds derived from the *Synechococcus* sp. biomass were identified via pyrolysis-gas chromatography-mass spectrometry (GC-MS), and 22 compounds were identified in this strain. The most abundant chemical compounds was (E)-octadec-11-enoic acid, with the peak area of 30.6%. Follow by tridecanoic acid, 12-methyl- and hexadecanoic acid, with a peak area of 23.26% and 18.23%, respectively. GC-MS analysis also identified indolizine, isoquinoline, 3,4-dihydro- and Phthalazine, 1-methyl-, as well as alkene and alkane from the strain. After the chemical toxicity test, 10 compounds were finally collected to do the further analysis. Then, network pharmacology and molecular docking were adopted to systematically study the potential anti-AD mechanism of these compounds. Based on the analysis, the 10 *Synechococcus-*derived active compounds could interact with 128 related anti-AD targets. Among them, epidermal growth factor receptor (EGFR), vascular endothelial growth factor A (VEGFA) and mitogen-activated protein kinase 3 (MAPK3) were the major targets. Furthermore, the compounds N-capric acid isopropyl ester, (E)-octadec-11-enoic acid, and 2H-Pyran-2,4(3H)-dione, dihydro-6-methyl- obtained higher degrees in the compounds-intersection targets network analysis, indicating these compounds may play more important role in the process of anti-AD. In addition, Kyoto Encyclopedia of Genes and Genomes (KEGG) enrichment analysis showed that these active compounds exert the anti-AD effects mainly through PI3K-Akt signaling pathway, neuroactive ligand-receptor interaction and ras signaling pathway. Our study identified *Synechococcus-*derived bioactive compounds have the potential for application to AD by targeting multiple targets and related pathways, which will provide a foundation for future research on applications of marine cyanobacteria in the functional drug industry.

## 1 Introduction

Recently, cyanobacteria (blue-green algae) have been accepted as one of the economic and viable sources with potential applications in various fields, including food, nutraceutical, therapeutics, pharmaceutical, cosmetics and personal care industries (Bourgade and Stensjö, 2022; Pathania et al., 2022; Bishoyi et al., 2023). They are an extremely diverse group of photosynthetic prokaryotes, owing to their adaptive capacity to tolerate extreme conditions, cyanobacteria are found in almost all the habitats of the Earth, from desert crusts to open ocean (Ferris et al., 1996; Nubel et al., 1999; Garcia-Pichel and Pringault, 2001; Gaysina et al., 2019). A variety of natural ingredients are reported to be found in cyanobacteria, including vitamins, proteins, polysaccharides, lipids, sterols, enzymes as well as other fine chemicals (Dahms et al., 2006; Abed et al., 2009; de Jesus Raposo et al., 2013; Hassan et al., 2022). The metabolic versatility of cyanobacteria lends them to bioengineering applications for sustainable production of various products (Stal, 2007; Xie et al., 2017; Momper et al., 2019; Liu et al., 2021). Especially, cyanobacteria derived bioactive compounds have a development potential in scientific research and great opportunity for drug discovery (Vijayakumar and Menakha, 2015; Sahoo et al., 2021). For example, apratoxin D isolated from cyanobacterium *Lyngbya* sp. has exhibited powerful cytotoxicity to human lung cancer cells (Gutierrez et al., 2008). Additionally, Symplocamide A, produced by marine cyanobacterium *Symploca* sp. has shown strong cytotoxicity to neuroblastoma as well as lung cancer cells (Linington et al., 2008). Cyanobacteria are also reported to be a rich source of omega-3 fatty acids, including eicosapentaenoic acid (EPA) and docosahexaenoic acid (DHA), which have the potential to prevent inflammatory and cardiovascular diseases (Sijtsma and de Swaaf, 2004; Udayan et al., 2017; Priyadarsini et al., 2022). Furthermore, cyanobacteria derived compounds could not only be applied directly as drugs, but also be used as potential lead compounds for new drug discovery (Tan L. T. 2007). For example, as potential anticancer drugs, curacin A from cyanobacteria *Lyngbya majuscule* and dolastatin 10 from cyanobacteria *Symploca sp.*, have been in preclinical and entered into clinical trials, respectively. Moreover, these components also served as drugs leading to the discovery of synthetic analogues, such as TZT-1027, compound 4, LU-103793, and ILX-651, usually holding improved pharmacological and pharmacokinetic properties for the treatment of multiple types of cancers (Uzair et al., 2012). Furthermore, cyanobacteria could also applied in the field of nanotechnology (Mandhata et al., 2022, Bishoyi et al., 2023). Sahoo et al. synthesized silver-nanoparticles (AgNPs) with cyanobacterium *Nostoc sphaeroides* and explored their antibacterial activity against bacterial strains that could lead urinary tract infection (Sahoo et al., 2021).

In the ocean, most species of picoplanktonic cyanobacteria currently known belong to two genera: *Prochlorococcus* and *Synechococcus* (Palenik et al., 2003). They are the most abundant photosynthetic organisms on Earth, numerically dominating most oceanic waters (Scanlan et al., 2009). Compared to *Prochlorococcus*, *Synechococcus* has a lower abundant in very oligotrophic waters, but has a broader global distribution, ranging from polar through temperate to tropical environments (Flombaum et al., 2013). For example, in some coastal areas, they could account for 20% of the local primary productivity (Li, 1994; Tai and Palenik, 2009). Due to the great deal of genetic and metabolic versatility within the *Synechococcus* sp., with strains probably adapted to complex and specific ecological environments (Scanlan et al., 2009; Bourgade and Stensjö, 2022). Besides, due to better adaptation to eutrophic environment, marine *Synechococcus* sp. is relatively easy to culture in the laboratory (Ahlgren and Rocap, 2006). Compared to *Prochlorococcus*, *Synechococcus* require less care and exhibit a faster growth rate, making them great candidates as bioproducers (Partensky et al., 1999; Yu J. et al., 2015; Ungerer et al., 2018). It has been reported that marine *Synechococcus* sp. contain multiple biologically active compounds, which include antibacterial, antifungal, antiviral, and cancer-fighting compounds (Martins et al., 2008; Costa et al., 2013; do Amaral et al., 2020; Srimongkol et al., 2023). Numbers of studies have indicated that they also have antioxidant and anti-inflammatory potential (de Morais et al., 2015; Gómez et al., 2023). For instance, Phycocyanin is a pigment found in blue-green algae, such as *Synechococcus* sp. (Hsieh-Lo et al., 2019). It has reported has an antioxidant activity and anti-inflammatory properties that works through the inhibition of histamine release (Romay et al., 1998; Bhat and Madyastha, 2001; Tan H. T. et al., 2022). As is well known, oxidative stress and chronic inflammation plays an important role in the progression of neurodegenerative diseases, such as Parkinson’s disease (PD), Alzheimer’s disease (AD), and multiple sclerosis (MS) (Pangestuti and Kim, 2011). As the various biologically potential of *Synechococcus*-derived active compounds, such as antioxidant and anti-inflammatory, do they also have anti-AD potential? At present, the knowledge on the anti-AD potential of the substances derived from marine *Synechococcus* sp. is still scarce, as well as the pharmacological mechanism.

AD, one of the major cause of dementia, is a degenerative brain disease characterized by the aggregates of extracellular β-amyloid (Aβ) plaques and intracellular tau protein, leading to neurofibrillary tangles, neuronal loss and cell death (Parihar and Hemnani, 2004; Citron, 2010). AD derived dementia is characterized by impairment in memory, language, problem-solving and other cognitive domains that affect a person’s ability to perform daily life activities (Kim K. et al., 2020). Until now, Acetylcholinesterase inhibitors (AChEIs) are remain the primary therapy for AD patients with mild-to-moderate cognitive impairment (Yu B. et al., 2022). However, these drugs do not have significant disease-modifying effects, but only provide symptomatic relief (Yeo-Teh and Tang, 2023). Hence, an efficient strategy is urgently needed for AD therapy, and a novel strategy is needed for anti-AD drugs discovery. Due to the diversity of species and metabolism, as well as unique advantages such as efficient photosynthesis and carbon sequestration, active compounds derived from microalgae provide us with new directions (Chen et al., 2022, Pathaniaet al. 2022). For example, a newly developed anti-AD drug‒sodium oligomannurarate, also called GV-971, was derived from marine algae, which can eventually accelerate Aβ clearance (Wang X. et al., 2019). Recently, network pharmacology strategy has proven to be powerful in explaining the molecular mechanisms of natural medicine, such as traditional Chinese medicine and marine natural products, which has complicated ingredients and multiple targets (Hopkins, 2007; Wang S. et al., 2022; Tikhonova et al., 2023). In addition, it could also serve as the basis for the novel drugs discovery. For example, Zeng et al. had used network pharmacology approach to uncover the key ingredients in Ginkgo Folium, as well as their anti-AD mechanisms (Zeng et al., 2021a).

**FIGURE 1:**
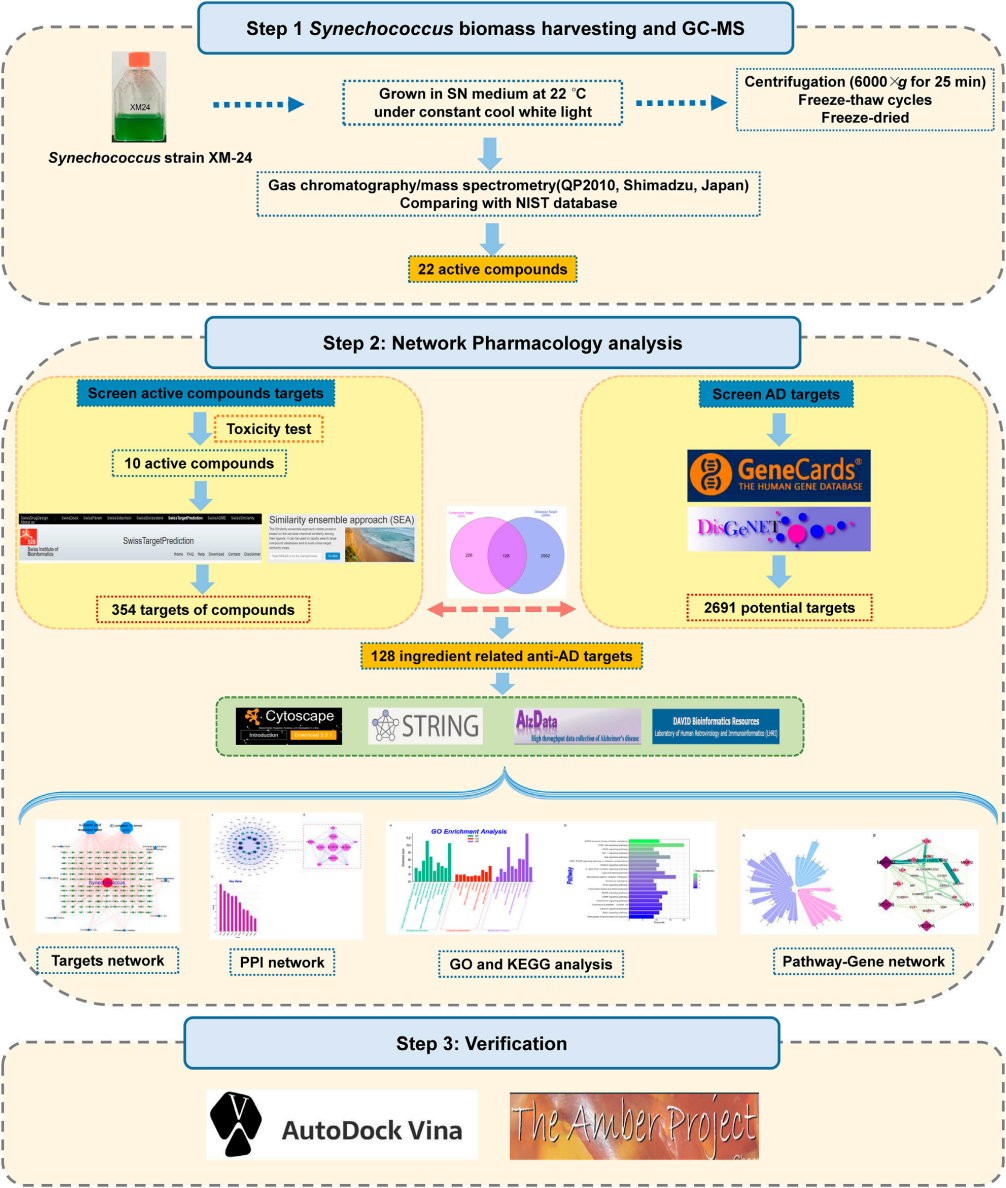
Flow chart of a network pharmacology-based strategy to investigate marine *Synechococcus* sp. derived compounds for application to Alzheimer’s disease.

In this study, we identified the organic compounds derived from marine cyanobacteria *Synechococcus* sp. XM-24 strain via pyrolysis-gas chromatography-mass spectrometry (GC-MS). Then, we evaluated the anti-AD potential of these compounds by applying network pharmacology strategy and molecular docking analysis. The study flow chart is shown in Figure 1. It is hoped that our study will allow further insights into the exploration of active compounds derived from marine cyanobacteria.

## 2 Materials and methods

### 2.1 *Synechococcus* culture and biomass harvesting

Marine *Synechococcus* sp. XM-24 was used in this study. *Synechococcus* sp. XM-24 was isolated from the coastal region near Xiamen Island, China, and belonged to clade CB5 in subcluster 5.2 (Zheng Q. et al., 2018). The strain were grown in the SN medium at 22°C under constant cool white light. The light intensity was 20–30 μE m^−2^ s^−1^. *Synechococcus* cells were collected via centrifugation (6000×*g* for 25 min) at the exponential phase (∼15 days). To harvest *Synechococcu*s-derived active compounds, the *Synechococcus* sp. XM-24 biomass was distributed in falcon-tube, and cells were subjected to freeze‒thaw cycles, then freeze‒dried, and kept frozen (–20°C) until further processing.

### 2.2 GC‒MS analysis

The pyrolysis-gas chromatography-mass spectrometry (GC‒MS) was applied to analysis the chemical composition of *Synechococcus* sp. XM-24 biomass as described by Yao Zhang et al. (Zhang et al., 2016). *Synechococcus* sp. cells were moved into a quartz tube. Then, the biomass were moistened with tetramethylammonium hydroxide solution (100μL, 25% in methanol). The sample was pyrolyzed using a single point pyrolyzer (Frontier Laboratories Ltd., Japan) at 550°C, interfaced directly to a GC‒MS (QP 2010, Shimadzu, Japan). About the stability of the temperature, the room temperature changed by 1°C, the change of the in-cylinder temperature was less than 0.01°C. The products were automatically injected into the gas chromatograph. Helium (99.999%) was used as a carrier gas. The flow rate was 1.8 ml min^−1^. The column temperature was set at 40°C for 3 min, and increased to 300°C at a rate of 10°C min^−1^ for 15 min. The mass spectrometer was operated at an ionization energy of 70 eV (EI). The mass range for detection was 25–500 m/z with a cycle time of 1s. Then, National Institute of Standards and Technology Mass Spectral Library database (NIST) was used for compound identification by comparing the obtained mass spectra with the database.

### 2.3 Potential targets of *Synechococcus*-derived active compounds

The chemical toxicity test was performed through PubChem databases (https://pubchem.ncbi.nlm.nih.gov/search/search.cgi), and all toxic compounds were eliminated. Then, the Swiss Target Prediction (https://www.swisstargetprediction.ch/) (Daina et al., 2019) and Search Server (SEA) (https://sea.bkslab.org/) (Keiser et al., 2007) were used to identify the potential targets of all these *Synechococcus*-derived active compounds.

### 2.4 Potential targets database of Anti-AD

GeneCards (https://www.genecards.org/) (Rebhan et al., 1998) and DisGeNET (https://www.disgenet.org/) (Pinero et al., 2017) were employed to screen potential genes, by setting “Alzheimer’s disease” and “anti-Alzheimer’s disease” as the keyword. Then, after further processing and removing the duplicate targets, the final targets related to anti-AD were collected. In addition, both of collected compound targets and anti-AD targets were imported into the InteractiVenn online platform (http://www.interactivenn.net/) to draw a Venn graph. Venn diagrams were employed to determine the intersection targets between *Synechococcus*-derived active compounds and anti-AD.

### 2.5 Active compound‒intersection target network construction

Cytoscape software was used to build a visualization network graph of active compound-intersection targets. Firstly, the obtained main active compounds and the intersection targets were imported into the Cytoscape 3.9.1 software. A visualization network diagram was generated from this software. Then, the Network Analyer plugin in Cytoscape 3.9.1 was used to analyze the topology parameters of this network. Based on degree, betweenness, and closeness, hub nodes were identified (Yu B. et al., 2022).

### 2.6 Protein-protein interaction network construction

The STRING database (https://string-db.org/) was used to construct a protein–protein interaction (PPI) network for the activities of *Synechococcus*-derived active compounds against AD. In this study, PPI can help us understand the functions and relationships of key target proteins (Athanasios et al., 2017). The STRING database can collect and integrate data about known and predicted protein‒protein associations, including both direct and indirect interactions, from many organisms (Szklarczyk et al., 2017). On STRING website, the main targets were imported into the “Multiple proteins” option, and “Homo sapiens” was selected in the organism query. And other parameters was established as the “default” criterion. Then, the visualized PPI network map was established using Cytoscape software (Shannon et al., 2003).

### 2.7 GO functional analysis and KEGG enrichment pathway analysis

To further explore the pathways of the anti-AD, the intersection targets were entered in the DAVID database (https://david.ncifcrf.gov/) (Huang da et al., 2009). Gene Ontology (GO) enrichment analysis is composed of biological process (BP), cellular component (CC), and molecular function (MF). Kyoto Encyclopedia of Genes and Genomes (KEGG) is a knowledge database which could be used to describe the interaction between different molecules and reveal known metabolic pathways (Chen J. et al., 2015). These two analysis helped to figure out the function of the core targets and the critical pathways of *Synechococcus*-derived active compounds against AD. The intersection targets data were imported into the DAVID database, and GO and KEGG analyses were performed on these intersection targets. Additionally, KEGG pathway enrichment analysis was also performed using ClueGO plug-in in Cytoscape software.

### 2.8 Bioinformatic analysis of intersection targets correlated with Aβ and Tau pathology

To study the pathological correlation between Synechococcus-derived ingredients with Aβ and tau in the treatment of AD, the intersection targets were imported into the AlzData database (http://www.alzdata.org/index.html) to perform the correlation analysis. The results were visualized through the Hiplot online platform (https://hiplot.com.cn/cloud-tool/drawing-tool/list). For convenience observation, the analysis were magnified by 100 times. The targets related to Aβ, tau and both of Aβ and tau were constructed PPI network using STRING database and Cytoscape software, to identify the core targets most correlated with Aβ and tau.

### 2.9 Molecular docking

The AutoDock software Vina was used to molecular docking simulation, revealing the binding modes and the strength between *Synechococcus*-derived active compounds and their protein targets (Morris and Lim-Wilby, 2008). The two-dimensional structures of the main active compounds of *Synechococcus* sp. XM-24 were collected through PubChem database. The 3D coordinates of KDR (PDB ID, 5EW3; resolution, 2.5 Å) and BIRC5 (PDB ID, 4AOI; resolution, 1.9 Å) were downloaded from the Protein Data Bank (http://www.rcsb.org/pdb/home/home.do) (Burley et al., 2021), and the chemical structures of the main active compounds were obtained from the TCMSP platform. The active pockets in the 3D structure of the proteins were identified by AutoDock Vina Tools, after removing the water molecules and small molecule ligands water molecules from the protein, and adding hydrogen. Then, using the prepared protein models and ligand structures, molecular docking was conducted through the AutoDock Vina software. The lower the score of the docking results, the stronger the binding ability. The molecular docking results were displayed in two-dimensional (2D) and three-dimensional (3D) graphics.

### 2.10 Molecular dynamics simulation

Proteins and compounds were parameterized using AMBER ff19SB and GAFF force fields, respectively (Wang et al., 2006; Tian et al., 2020). The system was solvated using an OPC water model and a truncated octahedral water box with a cutoff of 10 Å. Periodic boundary conditions (PBCs) were applied, and the system was neutralized. Non-bonded van der Waals interactions were calculated using the Lennard‒Jones 12–6 potential with a cutoff of 10 Å, while long-range electrostatic forces were treated using the PME algorithm. The SHAKE algorithm was used to constrain covalent bonds involving hydrogen atoms (Ryckaert et al., 1977). To eliminate steric clashes, heavy atoms were initially subjected to a constraint force of 10 kcal/(mol·Å) and underwent energy minimization using a combination of 2,500 steps of steepest descent and 2,500 steps of conjugate gradients. The system was then relaxed through 10,000 steps of steepest descent and 10,000 steps of conjugate gradient energy minimization. Subsequently, a 20 ps NVT simulation was performed to gradually heat the system to 300 K. This was followed by two equilibration phases: a 200 ps restrained heavy atoms NPT simulation and a 500 ps unrestrained NVT simulation. The temperature was controlled at 300 K using a Berendsen thermostat, and the pressure was maintained at 1 atm using a Monte Carlo barostat with coupling constants and relaxation times set as 1 ps. Finally, a 100 ns NVT simulation was carried out with a time step of 2 fs. RMSD and RMSF analyses were conducted using the CPPTRAJ module (Roe and Cheatham, 2013).

## 3 Results

### 3.1 GC-MS analysis

GC-MS analysis identified twenty-two chemical compounds from *Synechococcus* sp. XM-24 biomass. The identification of *Synechococcus* sp derived compounds is based on the mass spectra, including the retention time and peak area, and comparing with NIST database. All substances information are shown in Supplementary Figure S1 and Supplementary Table S1 in the Supplementary Material. The major types of compounds are fatty acids, alkene, alkane and esters. The most abundant chemical compound from *Synechococcus* sp. XM-24 was (E)-octadec-11-enoic acid, with the peak area of 30.6%. The second most abundant compound was tridecanoic acid, 12-methyl-, with a peak area of 23.26%. Follow by hexadecanoic acid, with a peak area of 18.23%. These three compounds together accounted for 72.09% of the peak area of the GC-MS. GC-MS analysis also identified indolizine, isoquinoline, 3,4-dihydro- and phthalazine, 1-methyl-, from the strain XM-24. In contrast, their proportions were not very high.

### 3.2 Potential targets of the main compounds derived from *Synechococcus* sp

We performed a chemical toxicity test on the twenty-two compounds though PubChem databases. The result showed that some components have cell toxicity and neurotoxicity, such as isoquinoline, 3,4-dihydro-, benzenepropionitrile, indolizin and so on. We did not do further analysis of these compounds in this study. Finally, ten compounds were collected as candidates to do the further analysis (Table 1). Then, by searching multiple online databases, including Swiss Target Prediction and SEA, 354 potential targets of the ten compounds were identified.

**TABLE 1:** Profile of the key compounds.

R.Time	Time	Area%	Entry	CAS	CompName	Smiles
7.147	7.147	1.92	7039	85825-79-2	2H-Pyran-2,4(3H)-dione, dihydro-6-methyl-	CC1CC(=O)CC(=O)O1
14.3	14.299	1.43	12,814	7045-71-8	Undecane, 2-methyl-	CCCCCCCCCC(C)C
10.443	10.445	1.36	3688	15870-10-7	1-Heptene, 2-methyl-	CCCCCC(=C)C
13.413	13.415	1.19	7987	5004-46-6	Phthalazine, 1-methyl-	CC1 = NN = CC2 = CC = CC = C12
16.747	16.746	1.08	19,392	2311-59-3	N-capric acid isopropyl ester	CCCCCCCCCC(=O)OC(C)C
9.283	9.284	0.93	4359	589–18-4	Benzenemethanol, 4-methyl-	CC1 = CC = C(C=C1)CO
13.293	13.293	0.87	10042	821-97-6	3-Undecene, (Z)-	CCCCCCCC = CCC
11.203	11.203	0.8	3526	14289-96-4	Allyl methallyl ether	CC(=C)COCC = C
14.237	14.236	0.8	10043	1002-68-2	3-Undecene, (E)-	CCCCCCCC = CCC
17.733	17.733	30.6	115401	693-72-1	(E)-octadec-11-enoic acid	CCCCCCC = CCCCCCCCCCC(=O)O

### 3.3 Anti-AD targets of main compounds derived from *Synechococcus* sp

**FIGURE 2:**
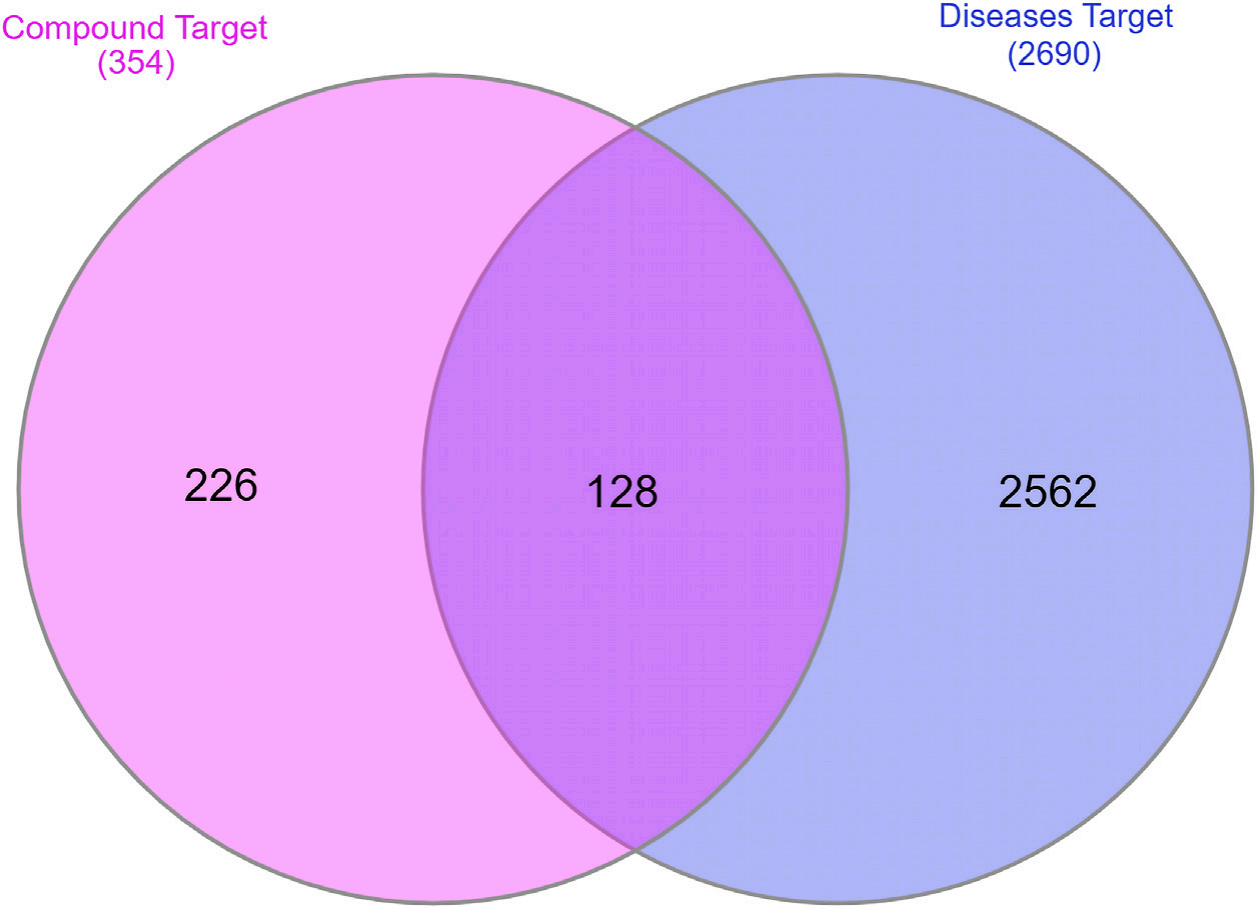
The intersecting targets between potential and AD-related targets. Pink: potential targets of active compounds; blue: targets of AD; purple: intersecting targets.

A total of 2,297 and 853 potential anti-AD targets were obtained by searching the GeneCards and DisGeNET databases, respectively. After removing duplicate targets, 2,691 potential anti-AD targets were finally collected. By employing the InteractiVenn online platform, the 354 predicted targets of the *Synechococcus*-derived active compounds and the 2,691 AD-related disease targets were used to construct the Venn diagram (Figure 2). The results showed that a total of 218 intersection targets of active compounds with potential anti-AD effects were obtained. These intersection targets were used to do the subsequent analysis.

### 3.4 Active compound-intersection target network

**FIGURE 3:**
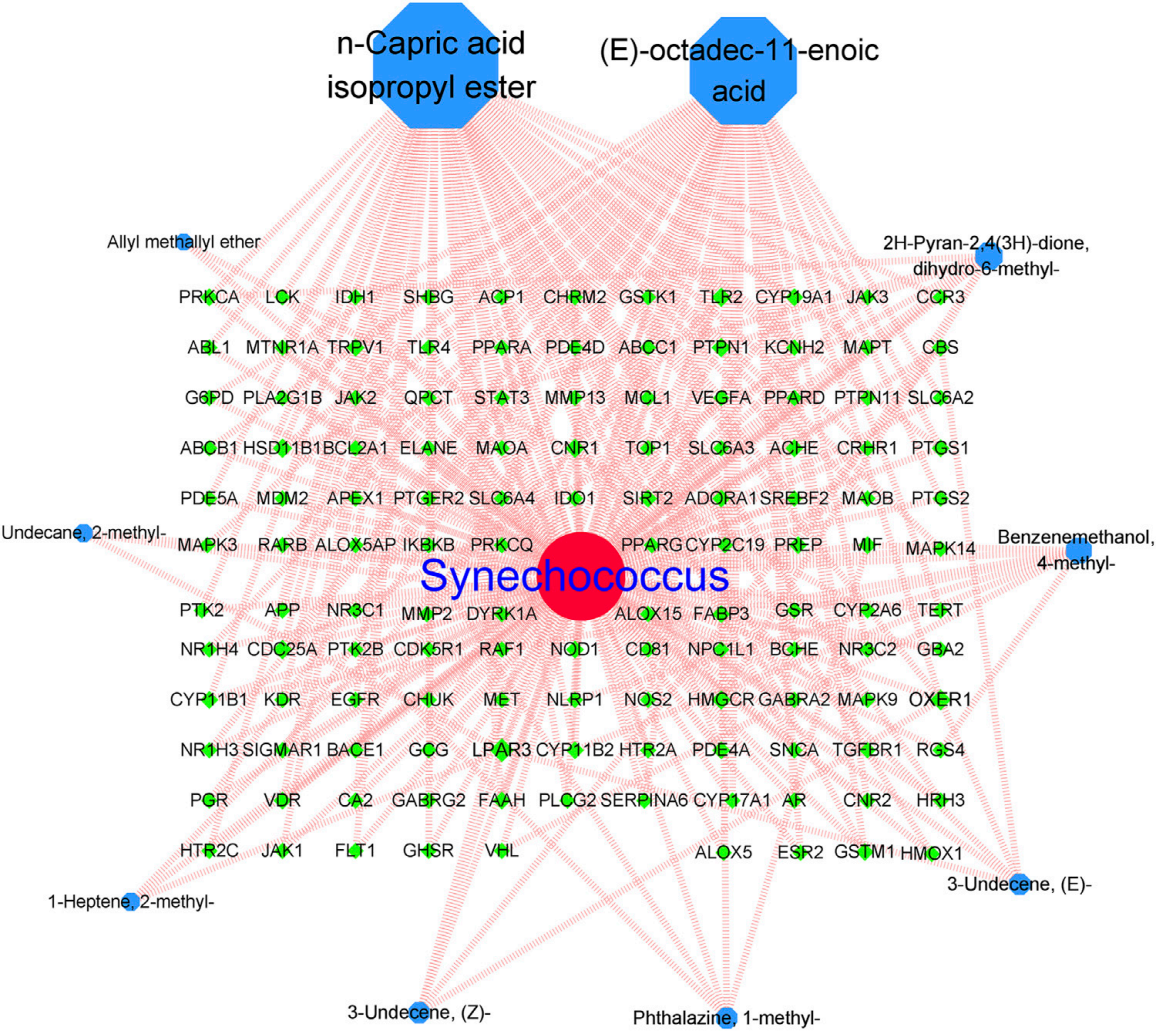
Active compounds-intersecting targets network. Blue: active compound nodes; green: targets of AD.

The network analysis results are shown in Figure 3. Finally, 10 *Synechococcus*-derived active compounds and 218 potential anti-AD targets were imported into the Cytoscape software for construction of visual network. network analysis revealed 139 nodes with 304 edges, with an average node degree of 4.374. Nodes represented the different active compounds and intersection targets. The connections between nodes represent the interactions between active compounds and their targets. As shown in Figure 3, most the active compounds could affected multiple targets, particularly compounds N-capric acid isopropyl ester and (E)-octadec-11-enoic acid. Moreover, the larger the node and the higher the degree value indicated more importance. In our study, compounds N-capric acid isopropyl ester (degree = 74), (E)-octadec-11-enoic acid (degree = 27), 2H-pyran-2,4(3H)-dione, and dihydro-6-methyl-(degree = 9) had larger nodes, indicating that they may play more important roles in the anti-AD process.

### 3.5 PPI results

**FIGURE 4:**
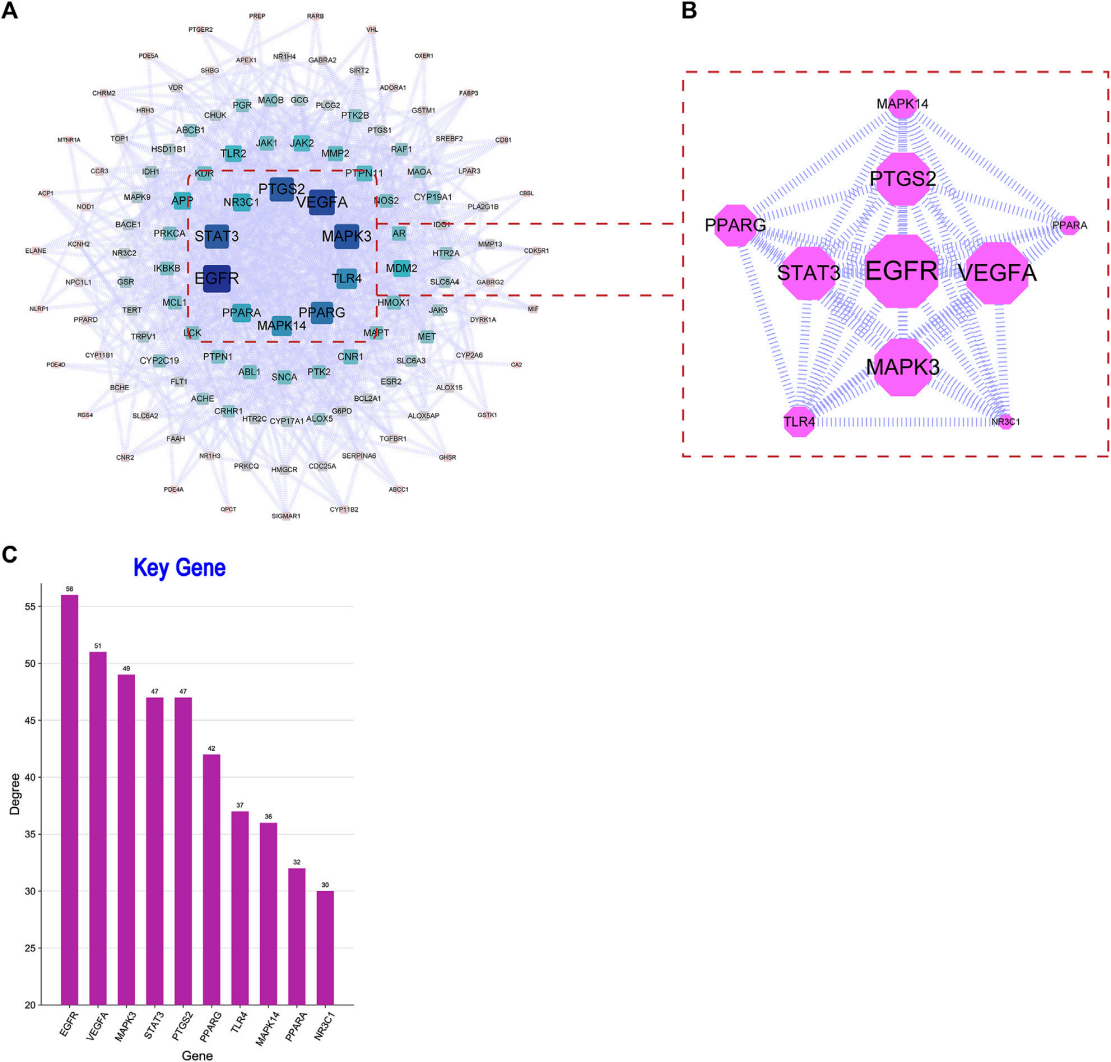
**(A)** PPI network. **(B)** The subnetwork of key targets. **(C)** Bar plot of key targets.

In this part of analysis, 218 intersection targets were imported into the STRING database. The exported data was in TSV format. After hiding the unrelated nodes, the data were imported into the Cytoscape software to bulid the PPI network. The visualized PPI results are shown in Figure 4. The PPI network contained 217 nodes and 853 edges, and the average degree of nodes was 13.433. In Figure 4A, the closer to the center of the concentric circles, the larger the nodes, and the darker the color. It also indicated that the closer to the center of the concentric circles, the more important the nodes. Taking the degree as the screening condition, the higher the degree was the more important of the targets in anti-AD process, as shown in Figures 4B, C. In the figure, epidermal growth factor receptor (EGFR), vascular endothelial growth factor A (VEGFA), mitogen-activated protein kinase 3 (MAPK3), signal transducer and activator of transcription 3 (STAT3) and prostaglandin-endoperoxide synthase 2 (PTGS2) were ranked high in degree value, indicating that they may play an important role in the process of anti-AD.

### 3.6 GO and KEGG enrichment pathway analysis

**FIGURE 5:**
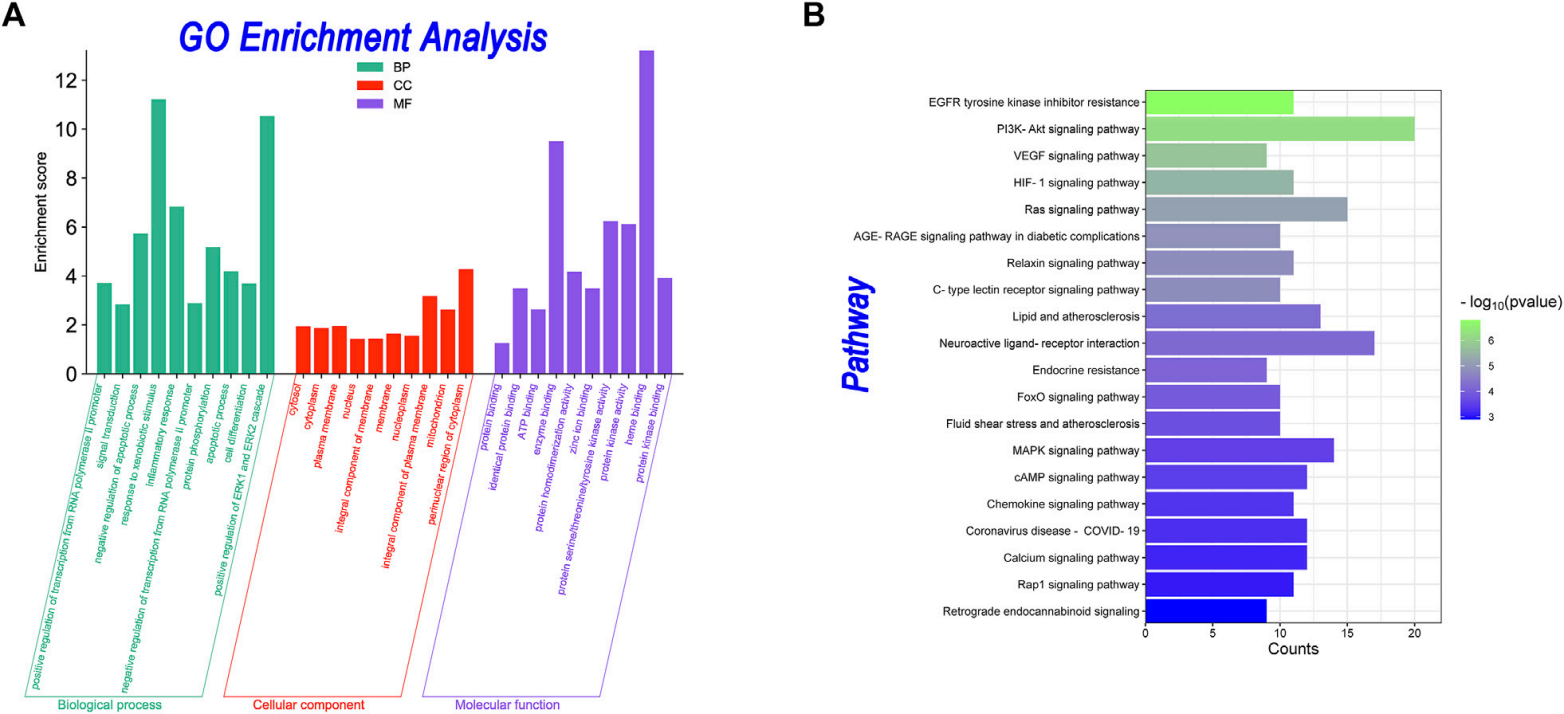
GO enrichment and KEGG pathway analyses. **(A)** Bar plot of GO enrichment analyses. The *x*-axis indicates the “biological process” in the GO. The *y*-axis indicates the enrichment score about these terms. **(B)** Bar plot of KEGG pathway enrichment analyses. The *x*-axis indicates the counts of the targets in each pathway. The *y*-axis indicates the main pathways.

**FIGURE 6:**
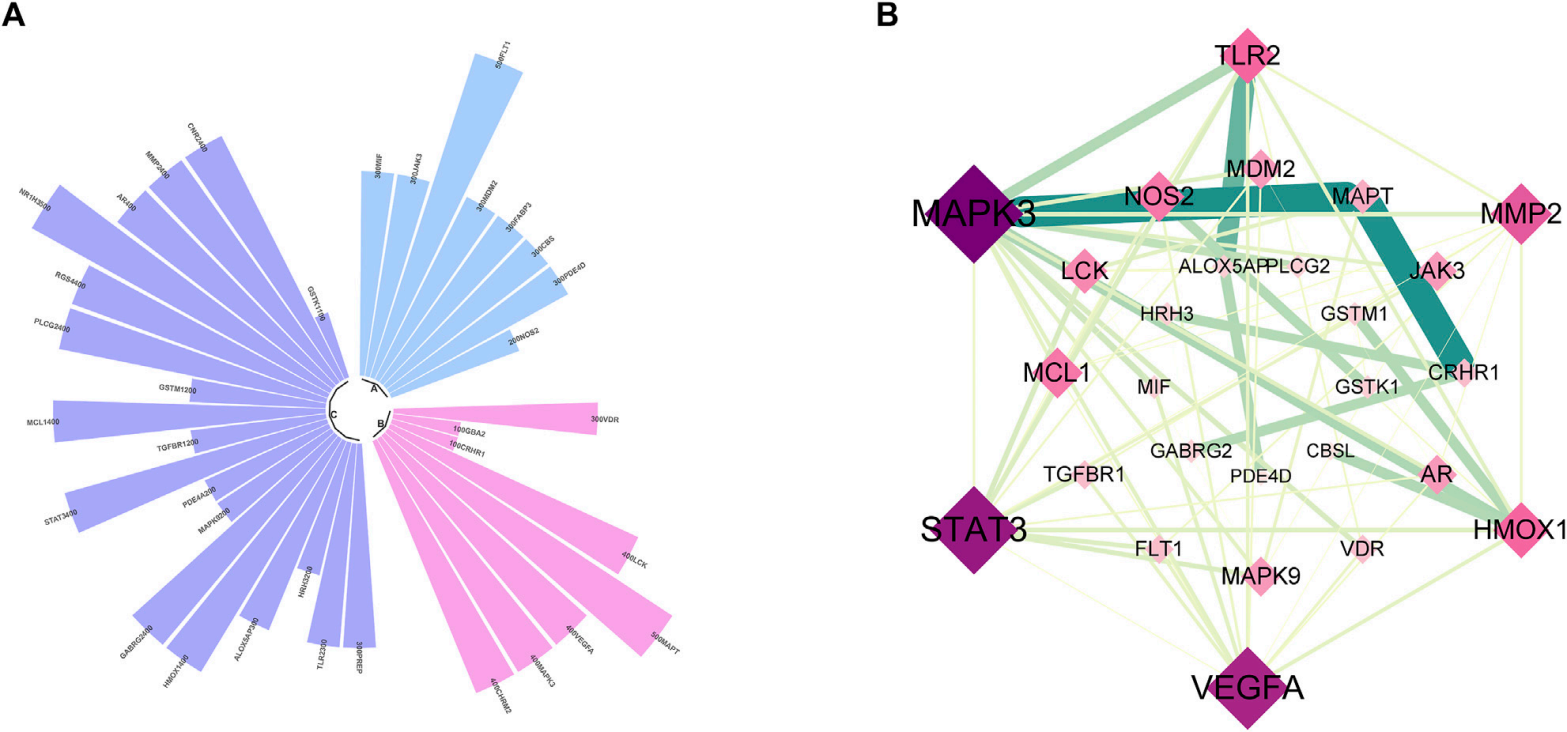
Bioinformatics analysis of active compounds targets associated with tau and Aβ pathology. **(A)** Donut plot. Blue: Aβ-related targets; pink: tau-related targets; purple: tau and Aβ-related targets. **(B)** PPI network.

In GO analysis, 288 GO enrichment entries were obtained, including 191 BP entries, 41 CC entries, 56 MF entries. In KEGG analysis, 86 KEGG signaling pathways were obtained. The results are shown in Figure 5. Figure 5A showed the GO analysis. For BP terms, the major enriched GO terms were positive regulation of transcription from the RNA polymerase II promoter (GO:0045944, count = 30), signal transduction (GO:0007165, count = 24), negative regulation of the apoptotic process (GO:0043066, count = 20), response to xenobiotic stimulus (GO:0009410, count = 19), and inflammatory response (GO:0006954, count = 19). The CC terms were cytosol (GO:0005829, count = 68), cytoplasm (GO:0005737, count = 67), plasma membrane (GO:0005886, count = 66), nucleus (GO:0005634, count = 54), and integral component of membrane (GO:0016021, count = 49). In MF, genes were mainly enriched in protein binding (GO:0005515, count = 109), identical protein binding (GO:0042802, count = 41), ATP binding (GO:0005524, count = 28), enzyme binding (GO:0019899, count = 25), and protein homodimerization activity (GO:0042803, count = 21). As shown in Figure 6B, the KEGG enrichment analysis revealed that the PI3K-Akt signaling pathway (hsa04151, count = 20), neuroactive ligand-receptor interaction (hsa04080, count = 17) and ras signaling pathway (hsa04014, count = 15), MAPK signaling pathway (hsa04010, count = 14), and Lipid and atherosclerosis (hsa05417, count = 13) were top-ranked, indicating that these pathways play a significant role in the anti-AD process.

### 3.7 Bioinformatic analysis of intersection targets associated with Aβ and tau pathology

To further explore the relationship between the potential targets of active compounds and the possible mechanisms of anti-AD, the intersection genes were imported into the AlzDate database. The results are shown in Figure 6. Among these targets (Figure 6A), eight were associated with Aβ, 8 were associated with tau, and 14 were associated with both Aβ and Tau. The 30 targets were imported into the STRING database to construct the PPI network, as shown in Figure 6B. A total of 27 nodes and 70 edges were found. After sorting by degree, the core targets included MAPK3 (degree = 16), STAT3 (degree = 14), VEGFA (degree = 13), MMP2 (degree = 9), TLR2 (degree = 8), and HMOX1 (degree = 8), indicating the multi-targeting of *Synechococcus* sp. against AD.

### 3.8 Molecular docking

**FIGURE 7:**
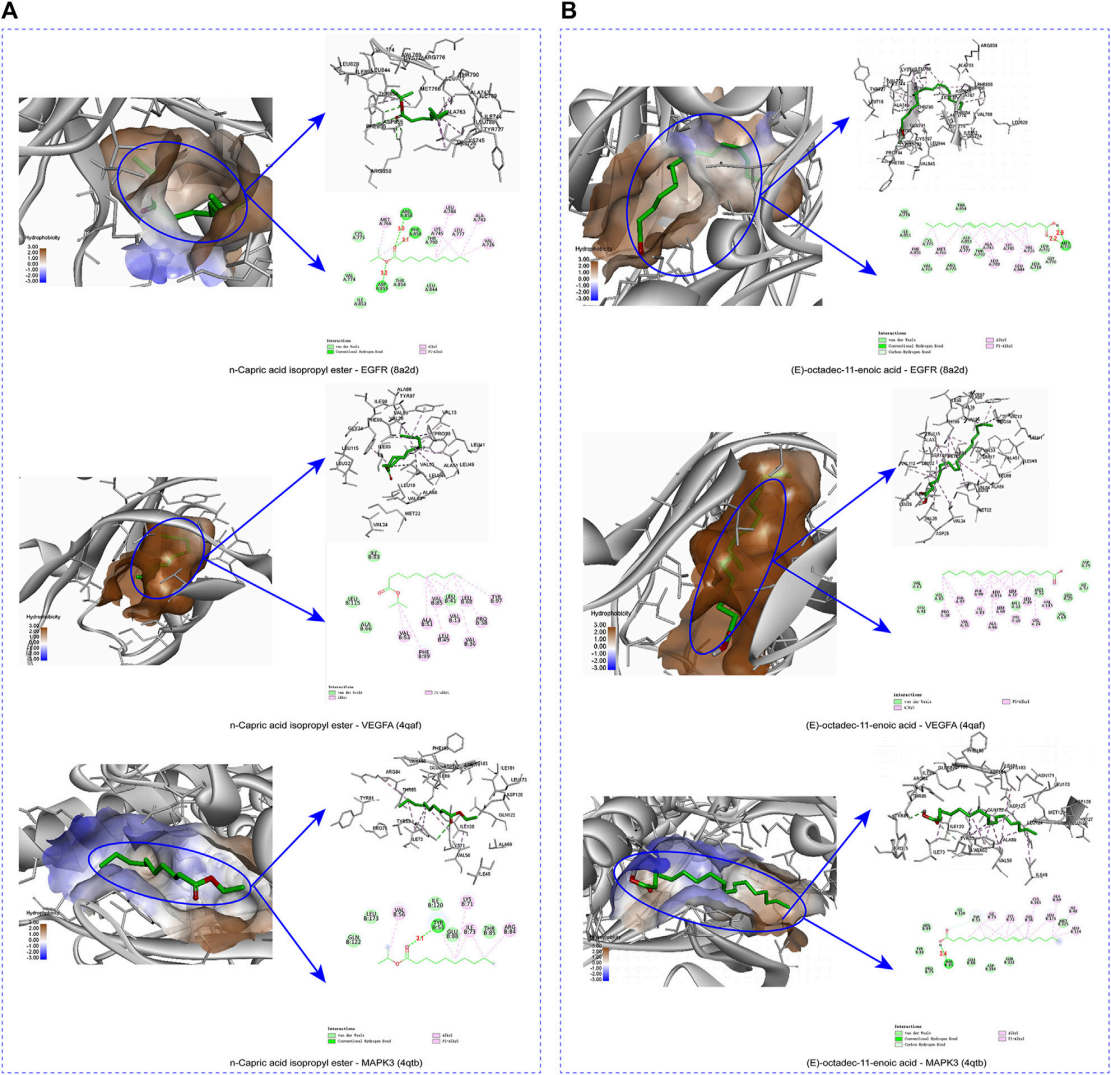
The protein-ligand of the docking simulation. **(A)** 3D and 2D maps of docking N-capric acid isopropyl ester with EGFR, VEGFA, and MAPK3. **(B)** 3D and 2D maps E-octadec-11-enoic acid of docking with EGFR, VEGFA and MAPK3.

To verify the binding ability between key components and key targets, molecular docking was performed using AutoDock Vina and the results were visualized using the visualization tool. N-capric acid isopropyl ester, (E)-octadec-11-enoic acid and EGFR (8A2D), VEGFA (4QAF) and MAPK3 (4QTB) molecules were docked as shown in Table 2. Among these targets, VEGFA (4QAF) was found to bind to N-capric acid isopropyl ester and (E)-octadec-11-enoic acid with scores of −6.3 and −7.2 kcal/mol, respectively, indicating that pocket VEGFA (4QAF) binds tightly to N-capric acid isopropyl ester, (E)-octadec-11-enoic acid and is stabilized by various interacting forces. Specifically, N-capric acid isopropyl ester potentially interacts with ARG858, ASP855, and PHE856 residues of EGFR (8A2D) through hydrogen bonding (Figure 7A). Among them, the distance between N-capric acid isopropyl ester and ARG858 was 3.0 Å, the distance to ASP855 was 3.2 Å, and the distance to PHE856 was 3.1 Å. N-capric acid isopropyl ester also potentially interacted with EGFR (8A2D) via van der waals, alkyl, and pi-alkyl. And N-capric acid isopropyl ester potentially interacted with VEGFA (4QAF) via van der waals, alkyl, and pi-alkyl. N-capric acid isopropyl ester binds to MAPK3 (4QTB) through the formation of hydrogen bond on TYP53 residue (Figure 7A), the distance between N-capric acid isopropyl ester and TYP53 is 3.1 Å; and the N-capric acid isopropyl ester potentially interacted with MAPK3 (4QTB) via van der waals, alkyl, and pi-alkyl. (E)-octadec-11-enoic acid potentially interacted with the MET793 residue of EGFR (8A2D) through hydrogen bonding (Figure 7B). The distances between (E)-octadec-11-enoic acid and MET793 were 2.9 and 2.2 Å. It also potentially interacted with EGFR (8A2D) via van der Waals, carbon-hydrogen bonding, alkyl, and pi-alkyl. (E)-octadec-11-enoic acid potentially interacted with VEGFA (4QAF) via van der waals, alkyl, and pi-alkyl. (E)-octadec-11-enoic acid bound to MAPK3(4QTB) through the formation of a hydrogen bond at the THR85 residue, which was separated from THR85 by a distance of 2.4 Å; and also potentially interacted with MAPK3(4QTB) via van der waals, carbon-hydrogen bond, alkyl, and pi-alkyl. These results suggest an important binding role between key components and key targets.

**TABLE 2:** Molecular docking parameters of N-capric acid isopropyl ester, (E)-octadec-11-enoic acid, and EGFR, VEGFA, and MAPK3.

Compounds (Ligand)	Targets (PDB ID)	Bingding energy (kJ/mol)	Center	Size
N-capric acid isopropyl ester	EGFR (8a2d)	−5.9	x = −16, y = −4, z = 30	x = 21, y = 21, z = 21
	VEGFA (4qaf)	−6.3	x = 13, y = 63, z = −1	x = 21, y = 21, z = 21
	MAPK3(4qtb)	−6.1	x = 54, y = 20, z = 85	x = 21, y = 21, z = 21
(E)-octadec-11-enoic acid	EGFR (8a2d)	−6.7	x = −16, y = −4, z = 30	x = 28, y = 28, z = 28
	VEGFA (4qaf)	−7.2	x = 13, y = 63, z = −1	x = 28, y = 28, z = 28
	MAPK3(4qtb)	−6.9	x = 54, y = 20, z = 85	x = 28, y = 28, z = 28

### 3.9 Molecular dynamics simulation

**FIGURE 8:**
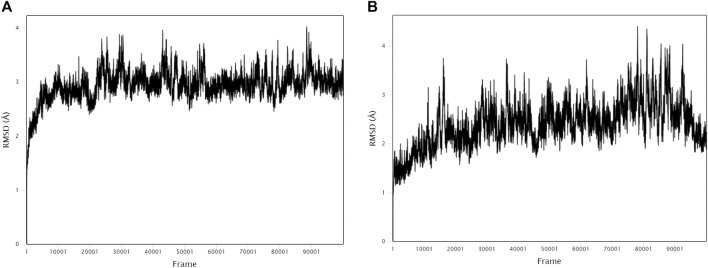
MD simulates RMSD trace values of protein-ligand complexes. VEGFA and **(A)** E-octadec-11-enoic acid, **(B)** N-capric acid isopropyl ester.

**FIGURE 9:**
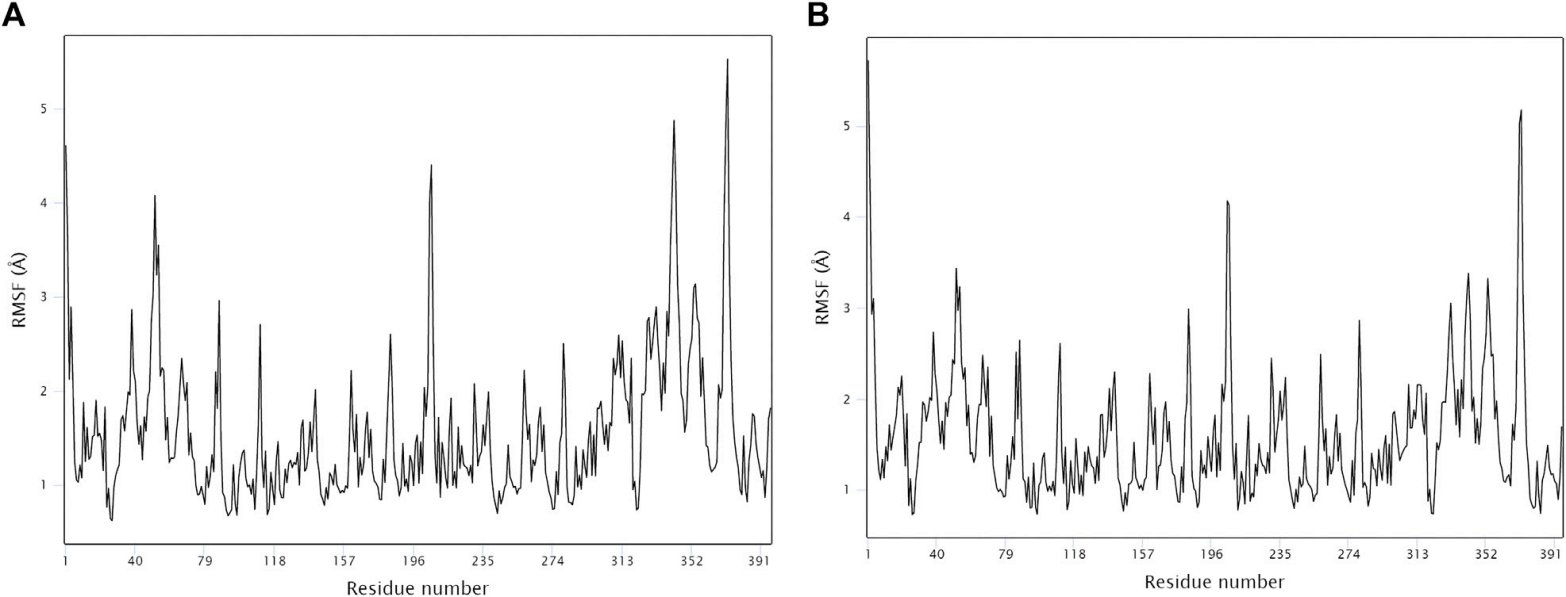
MD simulates RMSF trace values of protein-ligand complexes. VEGFA and **(A)** E-octadec-11-enoic acid. **(B)** N-capric acid isopropyl ester.

**FIGURE 10:**
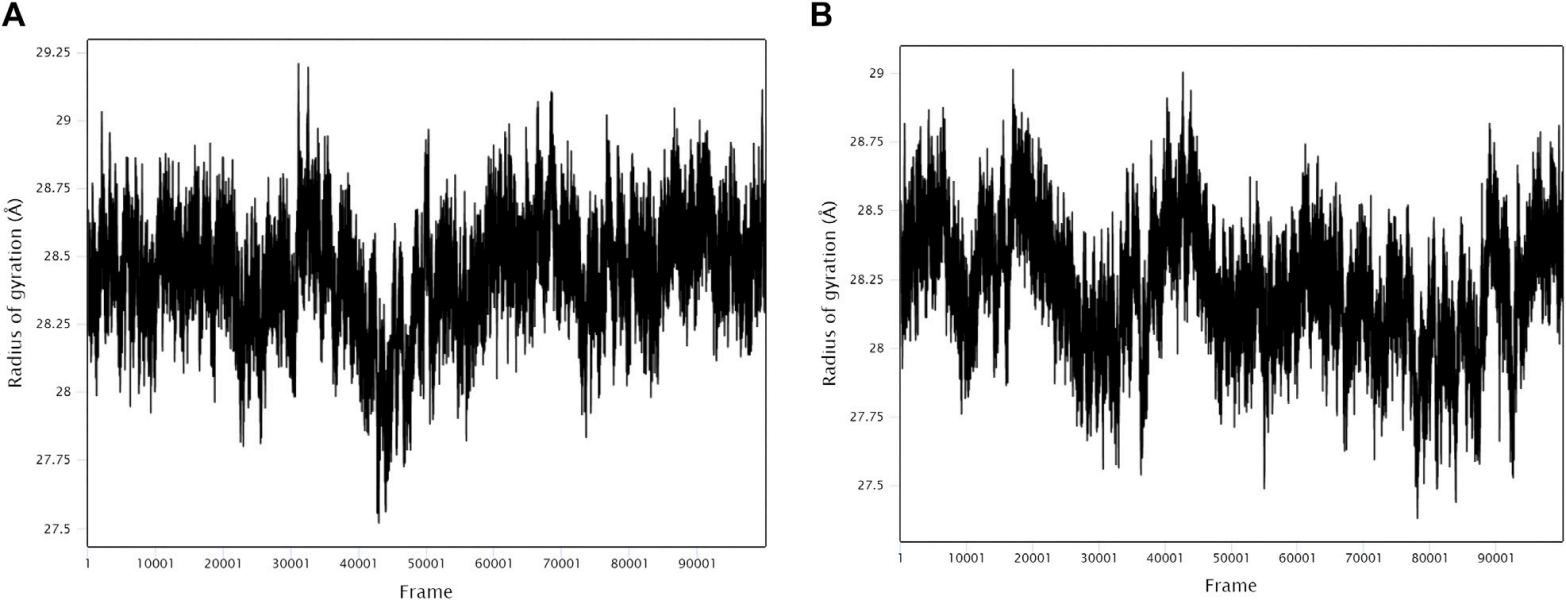
Radius of gyration (Rg) of protein-ligand complexes. VEGFA and **(A)** E-octadec-11-enoic acid, **(B)** N-capric acid isopropyl ester.

**FIGURE 11:**
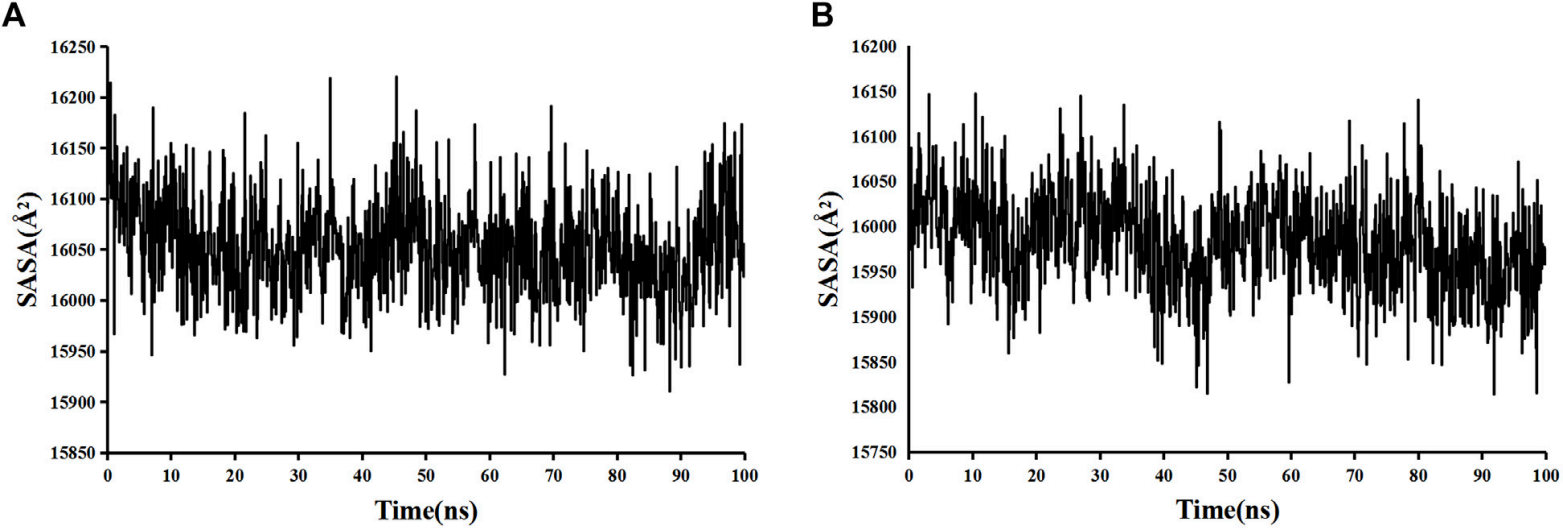
Solvent-accessible surface area (SASA) of protein-ligand complexes. VEGFA and **(A)** E-octadec-11-enoic acid, **(B)** N-capric acid isopropyl ester.

The stability and binding of many protein‒ligand complexes have been extensively studied using molecular dynamics (MD) simulations. Based on of molecular docking results, molecular dynamics simulations were performed to further evaluate the binding activity of VEGFA (4QAF)-(E)-octadec-11-enoic acid and VEGFA (4QAF)-N-capric acid isopropyl ester. In general, root mean square deviation (RMSD) analysis plays an important role in measuring the stability of proteins and ligands. Low RMSD values indicate a more stable protein-ligand complex. As shown in Figure 8, the RMSD values did not fluctuate significantly, indicating revealed that VEGFA-(E)-octadec-11-enoic acid and VEGFA-N-capric acid isopropyl ester could bind stably to VEGFA.In addition, the RMSF score was used to valuate protein flexibility across amino acid residues. Low RMSF values indicate decreased flexibility, whereas high RMSF values indicate increased flexibility. As shown in Figure 9, both VEGFA-(E)-octadec-11-enoic acid and VEGFA-N-capric acid isopropyl ester showed lower fluctuations in the protein residues represented by root mean square fluctuations. The majorfluctuations were observed near residues 51, 206, 342, and 472, indicating greater residue flexibility in these regions of the protein. As another indicator of stability, the radius of gyration (Rg) was also analyzed. The Rg value indicated that these two protein-ligand complexes did not fluctuate significantly during the MD simulation.At the end of the 100 ns simulation, the Rg values of the complexes were 28.5007Å (Figure 10A) and 28.1664Å (Figure 10B), respectively. In addition, we further explored the H-bond analysis calculated by accompanying molecular dynamics (MD) simulations. Consistent with the molecular docking results, the molecular dynamics simulation results also indicated that there were no hydrogen bonding interactions between the ligand and the target proteins. As shown in the SASA graph (Figure 11), the SASA values of VEGFA-(E)-octadec-11-enoic acid complexes are relatively stable, while the SASA values of VEGFA-N-capric acid isopropyl ester complexes show a slight downward trend from 0 to 100 ns.

## 4 Discussion

Many studies have demonstrated that the incidence of AD significantly increases with age (Dubois et al., 2021). With the exacerbation of the global aging trend, the number of AD patients is growing rapidly, and the demand for drugs to defeat AD is becoming increasingly urgent (Jeremic et al., 2021). For many years, marine products and their derivatives have been recognized as a rich source of therapeutic agents and structural diversity (Wolfender et al., 2019). Marine *Synechococcus* is an important genus of oceanic picocyanobacteria (Dore et al., 2022). They are widely distributed in the ocean, from the equator to the poles, showing an flexible adaptability to diverse environment (Callieri, 2017). Marine *Synechococcus* has many advantages such as easy culture and a high growth rate. Recent studies have shown that *Synechococcus* contain various active components, such as proteins, pigments, and fatty acids (Liu et al., 2021; Kothari et al., 2023). In particular, unsaturated fatty acids have been proven to have antioxidant, anti-inflammatory and anti-AD-related activities. However, so far, there have been few studies on the application of these components or their combinations in AD treatment. Consequently, network pharmacology, molecular docking, and molecular dynamics simulations were used to investigate the potential targets of *Synechococcus* sp. XM-24 for the treatment of AD.

Firstly, GC-MS was applied to identify the 22 constituents in the *Synechococcus* sp. XM-24. Among them, that (E)-octadec-11-enoic acid is the major constituent, while other volatile compounds such as indolizine, isoquinoline, alkene, and alkane, were also been founded. The current literature demonstrates that some compounds could have potential toxicity. Thus, by using multiple databases, we further selected out 10 compounds with higher safety for the further analysis. Subsequently, by constructing a network diagram of active compound-target-signaling pathways, we found that N-capric acid isopropyl ester and (E)-octadec-11-enoic acid had much higher degree values in the network analysis than other compounds. Structurally, these two compounds are medium-chain fatty acid esters and unsaturated fatty acids, respectively. Compound N-capric acid isopropyl ester was reported being found in *uraria picta Desv.*, a terrestrial plant (Mohan et al., 2020). However, its possible medicinal application was not exploring. Moreover, (E)-octadec-11-enoic acid are not commonly found in terrestrial photoautotroph. To date, very few existing literatures reports the anti-AD activity of these two compounds. There are some clues to suggest that these compounds are also promising therapeutic candidates to combat AD. Molecular docking results showed that both N-capric acid isopropyl ester and (E)-octadec-11-enoic acid had certain binding with EGFR, VEGFA, and MAPK3. At the current stage, research on medium-chain fatty acid esters is limited, and they are often used as food additives (Mayser, 2015). However, recent studies have also found that this type of compound has activities such as anti-psychotic and anti-bacterial activity (Koch et al., 2022). Trans-11-octadecenoic acid is a monounsaturated fatty acid that is also present in nuts and sea buckthorn fruit (Arruda et al., 2022). Pharmacological studies have shown that trans-11 vaccenic acid appears to have no health risks related to industrially produced trans fatty acids (Wang X. et al., 2016). In contrast, trans fatty acids are beneficial in the prevention of heart disease, obesity, and cancer (Lim et al., 2014; Wang et al., 2016). In addition, its derivatives have been found to have anti-oxidative stress activity. *In vitro* cell experiments showed that 10-Oxo-trans-11-octadecenoic acid (KetoC) can protect HepG2 cells from hydrogen peroxide-induced cytotoxicity. KetoC activates the Nrf2-ARE pathway to enhance *in vitro* and *in vivo* cellular antioxidant responses, further indicating that KetoC can prevent various diseases caused by oxidative stress (Furumoto et al., 2016). Based on existing studies, there is evidence of complex interactions between oxidative stress and the pathogenesis of Alzheimer’s disease (AD). In AD patients, elevated levels of protein oxidation, lipid peroxidation, and DNA damage products have been detected, suggesting the presence of oxidative damage (Ionescu-Tucker and Cotman, 2021; Afzal et al., 2021). Therefore, the active compounds found in *Synechococcus* sp. XM-24 hold potential in exerting anti-AD activity through the inhibition of oxidative stress.

Based on the intersection targets analysis, we found that these 10 active compounds mainly play anti-AD effects by acting on 128 core targets. Among them, EGFR, VEGFA, MAPK3, STAT3, and PTGS2 have higher degree values in the network analysis and have been considered the most likely targets to exert anti-AD effects. Moreover, numerous studies have shown that the accumulation of Aβ peptide, which are the main component of amyloid plaques, is the main factor in the pathogenesis of AD (Janus et al., 2000; Tanzi et al., 2004; Guo T. et al., 2020a). In addition, there is a close relationship between the occurrence of tau protein and AD (Mandelkow and Mandelkow, 1998; Kolarova et al., 2012). Therefore, we also analyzed the pathological correlation between these targets to Aβ and tau by using the Alzdatabase. The results showed that 30 targets among these 128 targets were significantly correlated with tau, Aβ, or both. Among them, MAPK3, STAT3, VEGFA, MMP2, and TLR2 had the highest degree values. Furthermore, STAT3, VEGFA, and MAPK3 were also the key targets in the PPI network. Prior studies have shown that the transcription factor Stat3 is a typical inducer of astrocyte proliferation and can be activated in AD model mice and human AD patients (Pekny et al., 2016; Escartin et al., 2021). *In vitro* and *in vivo* studies have demonstrated that the accumulation of tau can cause the inactivation of STAT3, which in turn inhibits the expression of N-methyl-d-aspartate receptors (NMDAR), and subsequently caused memory impairment (Wan et al., 2021). In addition, many recent studies have exhibited that the VEGF family plays a significant role in regulating angiogenesis, neurogenesis, and neuron survival (Rosenstein and Krum, 2004; Eichmann and Simons, 2012). The decrease in VEGF-A levels in cerebrospinal fluid is found to have been associated with the higher risk of developing AD and cognitive impairment (Hohman et al., 2015). Moreover, higher concentrations of VEGFA in cerebrospinal fluid were found to be associated with slower rates of hippocampal atrophy and cognitive decline, particularly in AD biomarker-positive adults (Sweeney et al., 2015). Furthermore, MAPK pathway is an important signaling pathway which closely related to cell proliferation, differentiation, and apoptosis (Guo Y. J. et al., 2020b). Through activation of the MAPK signaling pathway, could strongly influence the pathogenesis of AD, such as neuronal apoptosis β-secretase activity and γ-secretase activity (Lee et al., 2008; Kim E. K. and Choi, 2010). Previous studies have shown that stable AD patients treated with acetylcholinesterase inhibitors can further improve cognitive impairment when combined with cilostazol (Tai S. Y. et al., 2017). Based on this, Oguchi T. et al. confirmed through cell experiments that cilostazol can inhibit Aβ-induced SH-SY5Y cell neurotoxicity by inhibiting oxidative stress and the MAPK signaling pathway (Oguchi et al., 2017). According to the research of Tian Qing et al., the traditional Chinese medicine-Uncaria rhynchophylla can exert therapeutic effects on AD by acting on several key targets such as MAPK3 (Zeng et al., 2021b). Based on multiple clinical practice studies conducted in China and Japan, Huang-Lian-Jie-Du decoction (HLJDD) has significant neuroprotective effects. By employing network pharmacology strategies, Zheng et al. confirmed that HLJDD mainly exerts anti-AD effects by acting on multiple potential therapeutic targets, such as VEGFA, AKT1, TNF, YP53 and PTGS2 (Zheng R. et al., 2023). These studies indicate that VEGFA is strongly associated with AD.

In this study, we also conducted GO analysis and KEGG pathway enrichment analysis on potential target genes of active compounds against AD. The results indicated that the PI3K-Akt signaling pathway, Neuroactive ligand-receptor interaction, and Ras signaling pathway were the most significantly enriched pathways. Many studies have proved that the PI3K-Akt pathway can affect various major biological processes including cell proliferation, growth, and survival (Xu et al., 2020; Long et al., 2021). These pathophysiological processes are closely related to the occurrence of AD (Kumar and Bansal, 2022). Hence, the regulation of PI3K-Akt signaling may be helpful for the treatment of AD (Fakhri et al., 2021; Kumar and Bansal, 2022; Khezri et al., 2023). Fuhai Li et al. proposed that PI3K-Akt is a core neuroinflammatory signaling pathway that leads to chronic neurodegeneration (Li F. et al., 2021). Ruolan Li et al. used the clinical clinically used traditional Chinese medicine Yizhiren (AOF) as the research object. *In vitro* experiments indicated that and demonstrated through cell experiments that AOF can significantly increase the phosphorylation levels of PI3K and Akt proteins, thereby confirming that AOF can exert anti-AD effects by regulating the PI3K/Akt pathway (Li R. et al., 2022). The Neuroactive ligand-receptor interaction is also considered to be an important signaling pathway that associated with AD. According to the analysis of AD brain RNA expression chip dataset in the GEO database, the downregulation of SIRT3 mRNA in the brain tissue of AD patients may promote the progression of AD by affecting this pathway (Song et al., 2020). Zhou et al. found multiple potential AD therapeutic targets closely related to the neuroactive ligand-receptor interaction signaling pathway through the integration of phenotype screening data and multi-pharmacology network research (Zhou et al., 2023). In addition, according to the literature research, intervening in the Rho signaling pathway can effectively regulate the cascade reaction of tau and amyloid-beta in AD (Desale et al., 2021; Kumari et al., 2023). Plant- and marine-derived secondary metabolites are expected to treat multiple neurodegenerative diseases through the Ras/Raf/MAPKs pathway, which provides a new perspective for developing promising new multi-target anti-AD drugs (Gravandi et al., 2023).

As a result, *Synechococcus* sp. XM-24 highly likely execute anti-AD effects by regulates PI3K-Akt signaling, Neuroactive ligand-receptor interaction and Rho signaling pathway, etc., whereas its active compounds of by interacting with STAT3, VEGFA, and MAPK3. Additionally, we employed molecular docking and molecular dynamics simulations to further investigate the stability and interactions between key compounds and core target proteins. Favorable docking scores demonstrated a strong and efficient binding between these key compounds and core target proteins. Furthermore, during the 70ns MD simulation process, the conformation of the complex remained relatively unchanged, indicating a stable binding between the key active compounds and core target proteins. These results provide strong evidence for the potential therapeutic effects of key active compounds in *Synechococcus* sp. XM-24 against Alzheimer’s disease.

Moreover, it is also worth mentioning that marine cyanobacteria has advantages different from terrestrial organisms, such as rapid growth rate and easy availability (Rodolfi et al., 2009; Bhalamurugan et al., 2018). There, the application of bio-renewable compounds in cyanobacteria can be a valuable future research direction. Besides, since the growth of cyanobacteria may be affected by many factors, including temperature, nutrients, and accompanying bacteria and so on. By revealing the key anti-AD compounds of *Synechococcus* sp. XM-24, we can develop an ideal culture condition for better outcomes. In summary, in this study, we efficiently selected the most promising and drug-like small molecules from *Synechococcus* sp. XM-24 by using GC‒MS technology and network pharmacology research strategies. Then, the molecular virtual docking and molecular dynamic simulation results were confirmed that these active compounds and their combinations are expected to exert anti-AD effects by acting on key targets such as STAT3, VEGFA, and MAPK. In general, our study provides new research strategies and ideas for the study of *Synechococcus* in the field of anti-AD. However, in future studies, the anti-AD activity of these ingredients and their combinations *in vitro* and *in vivo* remains to be experimentally determined to confirm our hypothesis.

## 5 Conclusion

In this study, GC‒MS, network pharmacology analysis, and molecular docking were used to identify the active compounds, core targets, and signaling pathways of *Synechococcus* sp. XM-24 to explore its potential mechanisms in the treatment of AD.

A total of 22 active compounds were identified by GC-MS and 10 of them were further analyzed using network pharmacology analysis. The result show that compounds N-capric acid isopropyl ester and (E)-octadec-11-enoic acid obtained higher degrees in the network pharmacology analysis, indicating their higher potential in anti-AD process. Moreover, molecular docking showed favorable intermolecular hydrogen-bond formation between them and three core targets (EGFR, VEGFA, and MAPK3). It is expected that these results could provide a theoretical framework for the application of marine cyanobacteria in the treatment of AD. However, we are of the opinion that further studies are necessary in order to elucidate the specific targets and mechanisms behind them. Marine cyanobacteria was seems to be clearly an essential source of bioactive compounds and will continue to be a source of significant drug leads in the pharmaceutical industry. Furthermore, the application of advanced technology, e.g., multi-omics approach and nanotechnology, will provide us strong supporting on discovering novel active compounds and therapeutic method.

## Data Availability

The datasets presented in this study can be found in online repositories. The names of the repository/repositories and accession number(s) can be found in the article/Supplementary Material.

## Abbreviations

AChEIs, acetylcholinesterase inhibitors; AD, Alzheimer’s disease; AOF, Yizhiren; BP, biological process; CC, cellular component; EGFR, epidermal growth factor receptor; GC-MS, gas chromatography-mass spectrometry; GO, Gene Ontology; HLJDD, Huang-Lian-Jie-Du decoction; KEGG, Kyoto Encyclopedia of Genes and Genomes; KetoC, 10-Oxo-trans-11-octadecenoic acid; EPA, eicosapentaenoic acid; DHA, docosahexaenoic acid; Aβ, β-amyloid; MAPK3, mitogen-activated protein kinase 3; MF, molecular function; NFT, neurofibrillary tangles; NMDAR, N-methyl-d-aspartate receptors; PTGS2, prostaglandin‐endoperoxide synthase 2; PPI, protein–protein interaction; VEGFA, vascular endothelial growth factor A.
