# Supplementary material for: Network pharmacology‒based analysis of marine cyanobacteria derived bioactive compounds for application to Alzheimer’s disease
Source: Front Pharmacol. 2023 Oct 19;14:1249632. doi: 10.3389/fphar.2023.1249632 (PMC10620974; doi:10.3389/fphar.2023.1249632)
Supplement: Supplementary file 1 [file Table1.DOCX]

***Supplementary Material***

**Network pharmacology-based analysis of marine**

**Cyanobacteria derived bioactive compounds for application to Alzheimer's disease**

**Rui Xie†, Feng Chen†, Yixuan Ma†, Wen Hu, Qiang Zheng, Yi Wu*, Jinguo Cao***

*** Correspondence:**

Yi Wu: wuyi@gmu.edu.cn

Jinguo Cao: [jinguocao@gmu.edu.cn](mailto:jinguocao@gmu.edu.cn)

**1 Supplementary Figure and Table**

**1.1 Supplementary Figure**

**
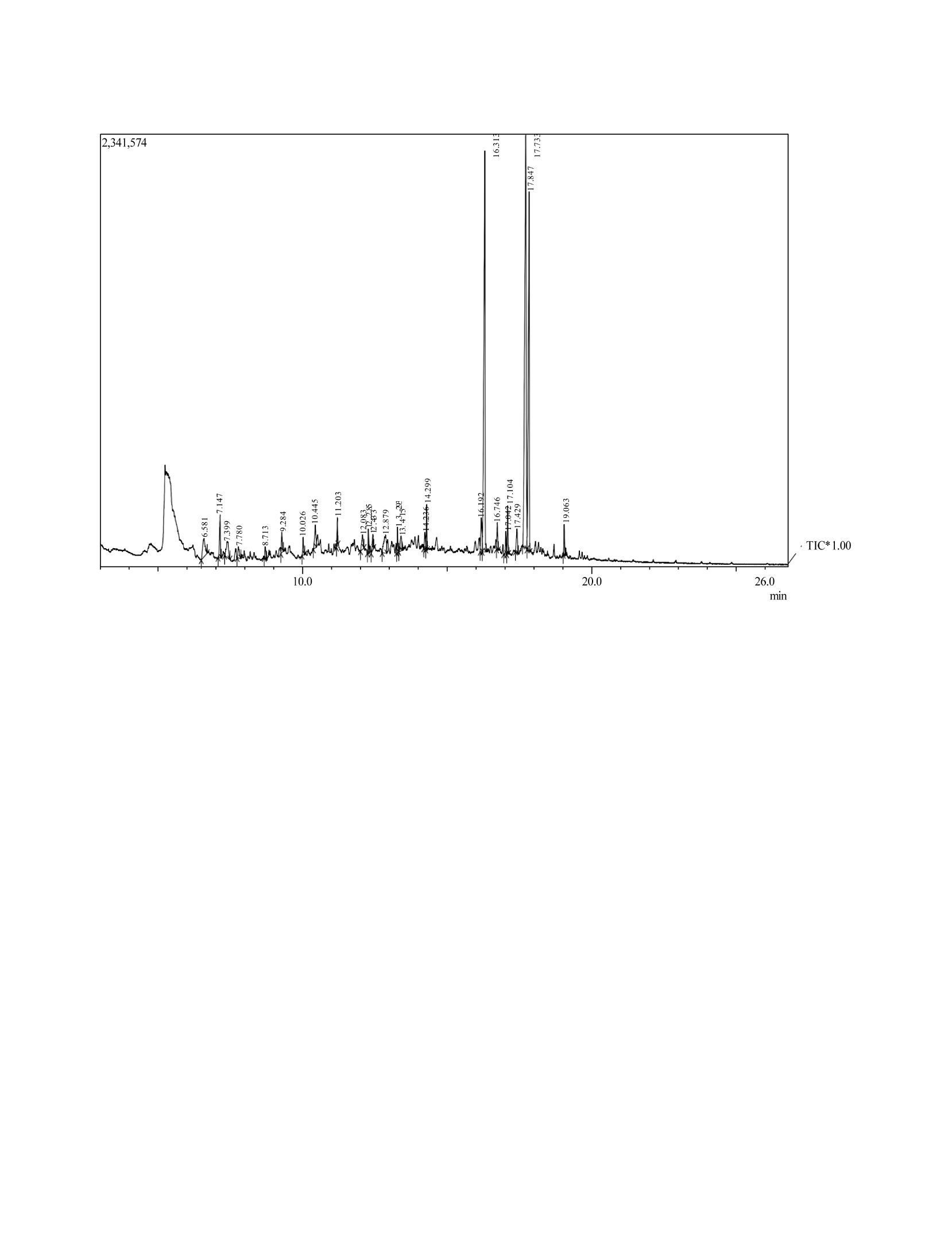
**

**Supplementary Figure S1.** GC-MS chromatogram of marine *Synechococcus* XM-24.

**1.2 Supplementary Table**

**1.2.1 Supplementary Table S1**

**Supplementary Table S1** Active compounds identified in *Synechococcus* sp. XM-24 biomass by GC–MS

| **NO.** | **Name** | **MV** | **Molecular Formula** | **CAS** | **SMILES** |
| --- | --- | --- | --- | --- | --- |
| 1 | Butanal, 3-hydroxy- | 88 | C_4_H_8_O_2_ | 107-89-1 | CC(CC=O)O |
| 2 | 2H-Pyran-2,4(3H)-dione, dihydro-6-methyl- | 128 | C_6_H_8_O_3_ | 85825-79-2 | CC1CC(=O)CC(=O)O1 |
| 3 | Styrene | 104 | C_8_H_8_ | 100-42-5 | C=CC1=CC=CC=C1 |
| 4 | Anisole | 108 | C_7_H_8_O | 100-66-3 | COC1=CC=CC=C1 |
| 5 | Allyl methallyl ether | 112 | C_7_H_12_O | 14289-96-4 | CC(=C)COCC=C |
| 6 | Benzenemethanol, 4-methyl- | 122 | C_8_H_10_O | 589-18-4 | CC1=CC=C(C=C1)CO |
| 7 | 1-Heptene, 2-methyl- | 112 | C_8_H_16_ | 15870-10-7 | CCCCCC(=C)C |
| 8 | Benzenepropionitrile | 131 | C_9_H_9_N | 645-59-0 | C1=CC=C(C=C1)CCC#N |
| 9 | 3-Undecene, (Z)- | 154 | C_11_H_22_ | 821-97-6 | CCCCCCCC=CCC |
| 10 | Isoquinoline, 3,4-dihydro- | 131 | C_9_H_9_N | 3230-65-7 | C1CN=CC2=CC=CC=C21 |
| 11 | Indolizin | 117 | C_8_H_7_N | 274-40-8 | C1=CC2=CC=CN2C=C1 |
| 12 | Phthalazine, 1-methyl- | 144 | C_9_H_8_N_2_ | 5004-46-6 | CC1=NN=CC2=CC=CC=C12 |
| 13 | 3-Undecene, (E)- | 154 | C_11_H_22_ | 1002-68-2 | CCCCCCCC=CCC |
| 14 | Undecane, 2-methyl- | 170 | C_12_H_26_ | 7045-71-8 | CCCCCCCCCC(C)C |
| 15 | 11-Octadecenoic acid, methyl ester | 296 | C_19_H_36_O_2_ | 52380-33-3 | CCCCCCC=CCCCCCCCCCC(=O)OC |
| 16 | Tridecanoic acid, 12-methyl-, methyl ester | 242 | C_15_H_30_O_2_ | 5129-58-8 | CC(C)CCCCCCCCCCC(=O)OC |
| 17 | n-Capric acid isopropyl ester | 214 | C_13_H_26_O_2_ | 2311-59-3 | CCCCCCCCCC(=O)OC(C)C |
| 18 | Tetradecanoic acid, 12-methyl-, methyl ester | 256 | C_16_H_32_O_2_ | 5129-66-8 | CCC(C)CCCCCCCCCCC(=O)OC |
| 19 | 1-NONYNE | 124 | C_9_H_16_ | 3452/9/3 | CCCCCCCC#C |
| 20 | 11-Tetradecen-1-ol, acetate, (Z)- | 254 | C_16_H_30_O_2_ | 20711-10-8 | CCC=CCCCCCCCCCCOC(=O)C |
| 21 | 11-Octadecenoic acid, methyl ester | 296 | C_19_H_36_O_2_ | 6198-58-9 | CCCCCCC=CCCCCCCCCCC(=O)OC |
| 22 | Hexadecanoic acid, methyl ester | 270 | C_17_H_34_O_2_ | 112-39-0 | CCCCCCCCCCCCCCCC(=O)OC |

**1.2.2 Supplementary Table S2**

**Supplementary Table S2 Databases information**

| **Datebases** | **Websites** | **Analysis Date** | **Reference** |
| --- | --- | --- | --- |
| PubChem databases | <https://pubchem.ncbi.nlm.nih.gov/search/search.cgi> | 2022.11.23 | (Kim et al. 2016) |
| Swiss Target Prediction | https://www.swisstargetprediction.ch/ | 2022.11.28 | (Daina et al. 2019) |
| Search Server (SEA) | https://sea.bkslab.org/ | 2022.11.28 | (Keiser et al. 2007) |
| GeneCards | https://www.genecards.org/ | 2023.3.27 | (Rebhan et al. 1998) |
| DisGeNET | <https://www.disgenet.org/> | 2023.3.27 | (Pinero et al. 2017) |
| InteractiVenn online platform | http://www.interactivenn.net/ | 2023.3.27 | (Heberle et al. 2015) |
| STRING database | https://string-db.org/ | 2023.3.30 | (Szklarczyk et al. 2017) |
| DAVID database | <https://david.ncifcrf.gov/> | 2023.4.18 | (Huang da et al. 2009) |
| AlzData database | <http://www.alzdata.org/index.html> | 2023.5.2 | (Sweeney et al. 2015) |
| Protein Data Bank | <http://www.rcsb.org/pdb/home/home.do> | 2023.5.23 | (Burley et al. 2021) |

**Reference**

Burley, S. K., Bhikadiya, C., Bi, C., Bittrich, S., Chen, L., Crichlow, G. V., et al. (2021). RCSB Protein Data Bank: powerful new tools for exploring 3D structures of biological macromolecules for basic and applied research and education in fundamental biology, biomedicine, biotechnology, bioengineering and energy sciences. *Nucleic Acids Res*. 49, D437-D451. doi: 10.1093/nar/gkaa1038

Daina, A., Michielin, O., and Zoete, V. (2019). SwissTargetPrediction: updated data and new features for efficient prediction of protein targets of small molecules. *Nucleic Acids Res*. 47, W357-W364. doi: 10.1093/nar/gkz382

Heberle, Henry, Meirelles, Gabriela Vaz, da Silva, Felipe R., Telles, Guilherme P., and Minghim, Rosane (2015). InteractiVenn: a web-based tool for the analysis of sets through Venn diagrams. *BMC Bioinformatics*. 16, 169. doi: 10.1186/s12859-015-0611-3

Huang da, W., Sherman, B. T., and Lempicki, R. A. (2009). Systematic and integrative analysis of large gene lists using DAVID bioinformatics resources. *Nat Protoc*. 4, 44-57. doi: 10.1038/nprot.2008.211

Keiser, M. J., Roth, B. L., Armbruster, B. N., Ernsberger, P., Irwin, J. J., and Shoichet, B. K. (2007). Relating protein pharmacology by ligand chemistry. *Nat Biotechnol*. 25, 197-206. doi: 10.1038/nbt1284

Kim, S., Thiessen, P. A., Bolton, E. E., Chen, J., Fu, G., Gindulyte, A., et al. (2016). PubChem Substance and Compound databases. *Nucleic Acids Res*. 44, D1202-1213. doi: 10.1093/nar/gkv951

Pinero, J., Bravo, A., Queralt-Rosinach, N., Gutierrez-Sacristan, A., Deu-Pons, J., Centeno, E., et al. (2017). DisGeNET: a comprehensive platform integrating information on human disease-associated genes and variants. *Nucleic Acids Res*. 45, D833-D839. doi: 10.1093/nar/gkw943

Rebhan, M., Chalifa-Caspi, V., Prilusky, J., and Lancet, D. (1998). GeneCards: a novel functional genomics compendium with automated data mining and query reformulation support. *Bioinformatics*. 14, 656-664. doi: 10.1093/bioinformatics/14.8.656

Sweeney, M. D., Sagare, A. P., and Zlokovic, B. V. (2015). Cerebrospinal fluid biomarkers of neurovascular dysfunction in mild dementia and Alzheimer's disease. *J Cereb Blood Flow Metab*. 35, 1055-1068. doi: 10.1038/jcbfm.2015.76

Szklarczyk, D., Morris, J. H., Cook, H., Kuhn, M., Wyder, S., Simonovic, M., et al. (2017). The STRING database in 2017: quality-controlled protein-protein association networks, made broadly accessible. *Nucleic Acids Res*. 45, D362-D368. doi: 10.1093/nar/gkw937
